# Cerebrovascular reactivity metrics as predictors of cognitive performance in healthy ageing: insights from transcranial colour-coded ultrasound

**DOI:** 10.1186/s13089-025-00445-1

**Published:** 2025-10-09

**Authors:** Joseph Amihere Ackah, Xiangyan Chen, Huixing Zeng, Jingxin Zhong, Jason Tsz Lok Chan, Michael Lung Cheung Lo, Jing Cai

**Affiliations:** 1 Department of Health Technology and Informatics, The Hong Kong Polytechnic University, Kowloon, Hong Kong China; 2 Division of Science, Engineering and Health Studies, College of Professional and Continuing Education, The Hong Kong Polytechnic University, Kowloon, Hong Kong SAR China; 3 Cerebrovascular Disease Center, Guangdong Province Traditional Medicine Hospital, Guangzhou, Guangdong China

**Keywords:** Cerebrovascular reactivity, Physiologic, Transcranial colour-coded Doppler ultrasound, Breath-holding, hyperventilation, cognitive impairment

## Abstract

**Introduction:**

This study was designed to investigate the utility of cerebrovascular reactivity (CVR) metrics, derived from transcranial colour-coded Doppler ultrasound (TCCD). Three main CVR metrics were examined as potential markers for cerebrovascular risk associated with mild cognitive impairment (MCI), a stage between normal cognition and dementia.

**Methods:**

We investigated 122 eligible, stroke-free, healthy, community-based Chinese adults (mean age, 65.34 ± 6.86 years). Cognitive performance was assessed using the validated Hong Kong version of the Montreal Cognitive Assessment. On a scale of 0–30, participants with low scores < 26 (modelled according to level of education) were designated to have a mild neurocognitive disorder or MCI. Following the measurement of cerebrovascular conductance (CVC) derived from cerebral blood flow and mean arterial pressure, three physiologic CVR metrics were assessed. The CVR assessments were based on restricted 30 s breath-holding, 60 s hyperventilation, and an unrestricted breath-holding index (BHI), respectively quantified using transcranial colour-coded Doppler ultrasound. The predictabilities and associations between CVR metrics, haemodynamic parameters, and cognitive performance were statistically investigated.

**Results:**

Using TCCD, BHI emerged as the most accurate and robust metric of CVR for predicting mild cognitive disorders [AUC 0.827 (95% CI 0.725, 0.930)] and independently predicted overall cognitive performance, highlighting its clinical value for early identification of at-risk individuals. The three CVR metrics outperformed CVC in predicting mild cognitive impairment and were distinctively correlated. Although CVR measures by breath-holding and BHI were closely related (r = 0.704, 95% CI 0.598, 0.786, p < 0.001), Bland–Altman analysis revealed that they are not interchangeable, indicating the importance of metric selection for accurate cerebrovascular assessment.

**Conclusion:**

The BHI, derived from simple and clinically tolerable methods, demonstrates clear potential to enhance the prediction and early identification of vascular cognitive impairment in healthy adults. By leveraging insights from cerebral haemodynamics, TCCD-based cerebrovascular risk screening may enable more effective and targeted interventions, ultimately contributing to better long-term cognitive health outcomes.

**Graphical Abstract:**

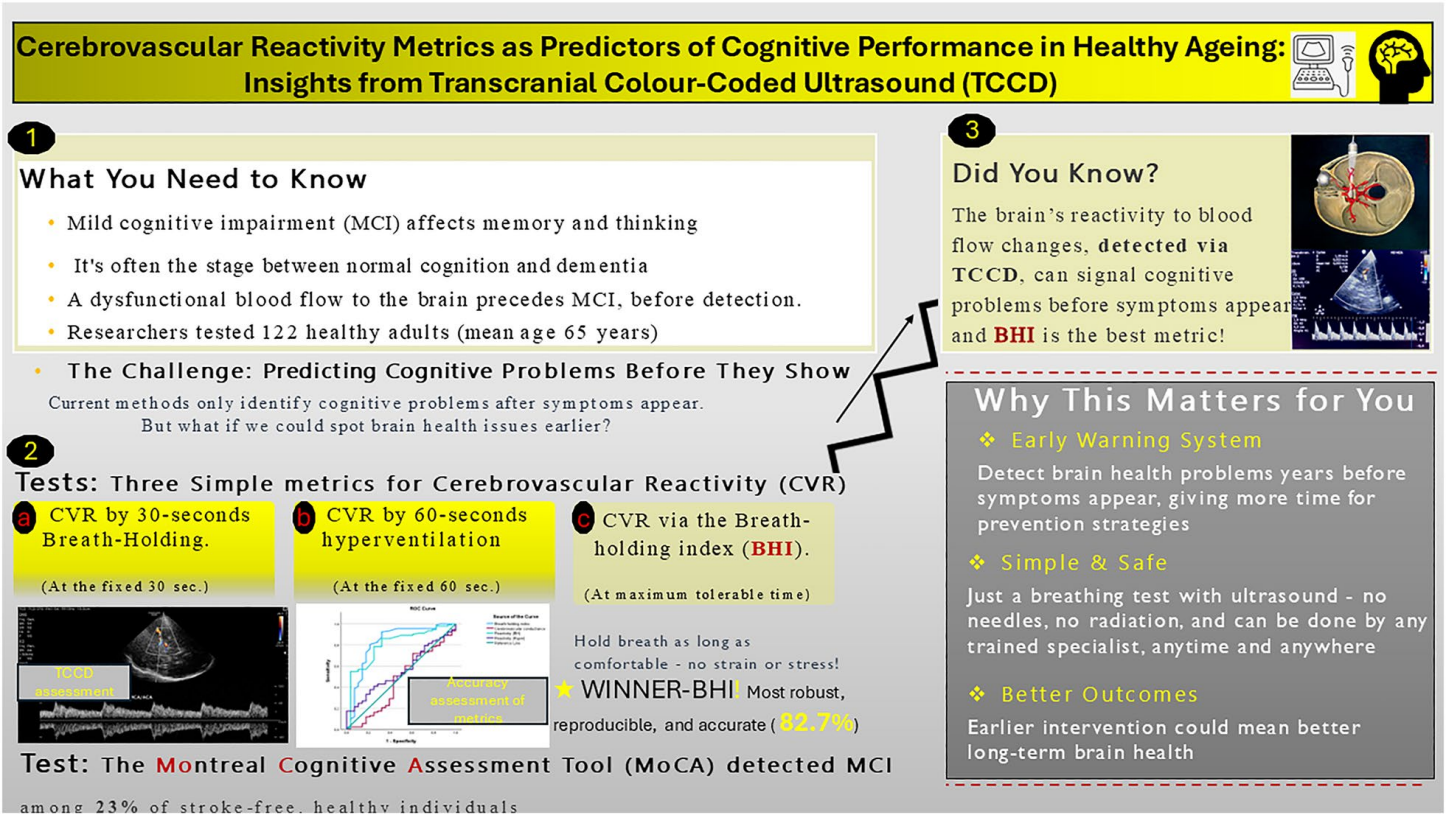

## Introduction

Cerebrovascular reactivity (CVR), commonly referred to as vasomotor reactivity, is a pivotal measure of the brain's capacity to regulate blood flow and its haemodynamic response to various metabolic demands [1, 2]. CVR is shown to play a vital role in cerebral haemodynamics, and the impairment of CVR is a recognised marker of ageing-related cerebrovascular dysfunction with consequent cognitive implications [2, 3]. As a measure of cerebrovascular function, different metrics of CVR could be distinctively influenced by haemodynamic vascular parameters, including cerebrovascular conductance and cerebral blood flow velocity [4, 5]. Various modalities such as single photon emission computed tomography (SPECT), positron emission tomography (PET), magnetic resonance imaging (MRI), as well as computed tomography (CT) perfusion, xenon CT, and near-infrared spectroscopy, have been employed to explore cerebrovascular reactivity, and each comes with unique limitations [6–8]. Notable challenges include exposure to ionising radiation [9], sophisticated modalities with complex applications, limited depth penetration and poor spatial resolution [10, 11], suboptimal functional data, and longer scanning time [12]. These challenges impede the clinical applicability of these modalities for screening or diagnostic purposes. In contrast, transcranial Doppler (TCD) and transcranial colour-coded Doppler (TCCD) ultrasound offer promising alternatives. Transcranial ultrasound imaging is non-invasive, cost-effective, and widely accessible, and its capability to reliably assess haemodynamic parameters indicates significant potential for evaluating CVR [13, 14].

Traditional transcranial Doppler methods for evaluating CVR involve inducing a transient vasodilatory stimulus and measuring the resulting changes in blood flow velocity relative to arterial carbon dioxide end-tidal pressure (pCO2), which reflects the vasomotor capacity of the subject's cerebrovascular function [15, 16]. For decades, carbon dioxide gas and acetazolamide have been used as vasodilatory stimuli to assess the vasomotor reactivity in the major cerebral vessels [7, 9, 17]. However, these methods are not without risks. Specifically, carbon dioxide inhalation is reported to cause shortness of breath and anxiety, especially in older patients with chronic lung disease, and it may lead to complications like respiratory acidosis [2]. Similarly, acetazolamide administration may cause perioral dysesthesia, arterial hypertension, headaches, and nausea and can even trigger counterproductive hyperventilation [18]. In response to these limitations, researchers have explored safer and more accessible methods. Notably, breath-holding and hyperventilation techniques have emerged as promising physiological approaches for assessing CVR [19, 20]. These methods offer a more sensitive and safer alternative for inducing vasodilatory hypercapnia and hypocapnia, respectively, reducing the risks associated with carbon dioxide inhalation and acetazolamide [19, 21]. It is important to note that CVR metrics based on physiologic methods are quantified as CVR by breath-holding, CVR by hyperventilation and a third metric known as the breath-holding index (BHI) [19, 20]. CVR by breath-holding measures the percentage change in cerebral blood flow velocity in response to increased CO_2_ levels during a voluntary breath hold; however, it does not account for the duration of the breath hold in its calculation. In contrast, the BHI is a specific CVR metric that quantifies the percentage change in cerebral blood flow velocity per second of breath-hold duration, thereby incorporating time as a critical factor. Although both CVR by breath-holding and BHI are assessed via similar breath-holding approaches, they are quantified differently, and it is currently unclear whether the two metrics are interchangeable. On the other hand, CVR by hyperventilation quantifies the blood flow response to decreased CO_2_ levels induced by hyperventilation. Although these three metrics offer unique insights into cerebrovascular health, the applicability of each metric in aiding clinical assessment and screening for ageing-related cognitive impairment has not been distinctly delineated [6].

To fully understand the clinical significance of cerebral haemodynamics in cerebrovascular health, it is necessary to examine how these CVR metrics relate not just to cognition but also to other haemodynamic factors, such as cerebrovascular conductance. Cerebrovascular conductance is a measure of the capacity of blood vessels to conduct blood flow, and it is influenced by both vascular tone and blood pressure. To date, the correlational differences and the reliabilities between the three distinct CVR metrics and cerebrovascular conductance as predictors of cognitive performance have not been systematically compared. We posit that these physiologically derived CVR metrics may differ meaningfully in their association with cerebrovascular conductance and cognition. Directly contrasting the predictive value of these metrics could clarify their respective roles in understanding cerebral haemodynamics and the mechanisms underlying vascular dysfunction and cognitive impairment with ageing.

In light of these considerations, this study was designed to investigate the utility of TCCD-derived CVR metrics as potential markers for assessing the cerebrovascular risk of mild cognitive impairment. The primary endpoint was to evaluate the predictive values of CVR measures—breath-holding, hyperventilation, and the BHI—in relation to global cognitive performance. To achieve this goal and contextualise CVR metrics with cerebral blood flow dynamics, the study pursued three objectives. First, it determined whether CVR by breath-holding, hyperventilation, and BHI correlate with each other. Second, it examined the differential associations between CVR metrics and other haemodynamic parameters, including cerebrovascular conductance. Third, it compared how cerebrovascular conductance and CVR metrics predict overall cognitive performance.

## Results

### Participant’s characteristics

One hundred and fifty (150) healthy participants from our ongoing community-based cohort volunteered for this study. Twenty-four (16.0%) were excluded due to a poor transtemporal window; four (2.7%) were unable to perform the breath-holding and hyperventilation manoeuvres. No participant demonstrated overt structural brain lesions on MRI that met exclusion criteria. Among 122 eligible, stroke-free participants (mean age, 65.34 ± 6.86 years), 66 (54.1%) were men, 34 (27.9%) had hypertension, 12 (9.8%) had diabetes, 49 (40.2%) showed hyperlipidaemia, 5 (4.1%) were active smokers, and 11 (9.0%) consumed alcohol. None had a history of stroke, neurodegenerative disease, or used vasodilatory drugs. Measures of cognitive performance, breath-holding duration, cerebrovascular conductance, and CVR metrics—breath-holding, hyperventilation, and BHI—were comparable between males and females. There were 94 (77%) cognitively normal participants and 28 (23%) with mild cognitive impairment. Clinical data, haemodynamics, and cognitive outcomes are summarised in Table 1.

**Table 1:** Participant’s characteristics

	Population (n = 122)	Male (n = 66)	Female (n = 56)	P value
Age (years)	65.34 ± 6.86	65.86 ± 6.71	64.71 ± 7.05	0.359
BMI (kg/m2)	23.44 ± 3.10	24.18 ± 3.10	22.56 ± 2.89	0.004
SBP (mmHg)	130.04 ± 16.94	133.76 ± 16.22	125.66 ± 16.85	0.008
DBP (mmHg)	79.22 ± 10.86	82.26 ± 10.19	75.64 ± 10.60	< 0.001
PR (bpm)	71.82 ± 10.37	72.32 ± 11.60	71.23 ± 8.77	0.566
MAP (mmHg)	96.16 ± 12.04	99.42 ± 11.52	92.32 ± 11.59	< 0.001
MCA velocity (cm/s)	46.53 ± 9.68	46.32 ± 8.72	46.78 ± 10.77	0.793
MCA RI	0.59 ± 0.07	0.58 ± 0.07	0.59 ± 0.07	0.758
MCA PI	0.93 ± 0.18	0.93 ± 0.19	0.93 ± 0.17	0.898
Reactivity (BH) (%)	46.35 ± 7.73	46.57 ± 6.32	46.09 ± 9.17	0.734
Reactivity (Hyper) (%)	27.32 ± 15.99	26.56 ± 12.76	28.19 ± 19.16	0.577
BHI (s^−1^)	1.09 ± 0.17	1.09 ± 0.14	1.09 ± 0.20	0.841
Conductance (cms^−1^/mmHg)	0.49 ± 0.13	0.47 ± 0.12	0.52 ± 0.15	0.066
Cognitive performance	24.80 ± 3.43	25.03 ± 2.68	24.52 ± 2.16	0.414
Max. breath-holding (s)	42.81 ± 6.87	42.88 ± 4.02	42.72 ± 9.20	0.900

Cerebrovascular conductance (cm/s/mmHg). The p-values for continuous variables, expressed as mean ± standard deviation (x̄ ± SD), were obtained using t-tests

BMI: body mass index; SBP: systolic blood pressure; DBP: diastolic blood pressure; PR: pulse rate; MAP: mean arterial pressure, MCA: middle cerebral artery; RI: resistive index; PI: pulsatility index; BH: breath-holding (%); hyper: hyperventilation (%); BHI: breath-holding index; s^−1^: per second

### CVR metrics and haemodynamic parameters

**Fig. 1:**
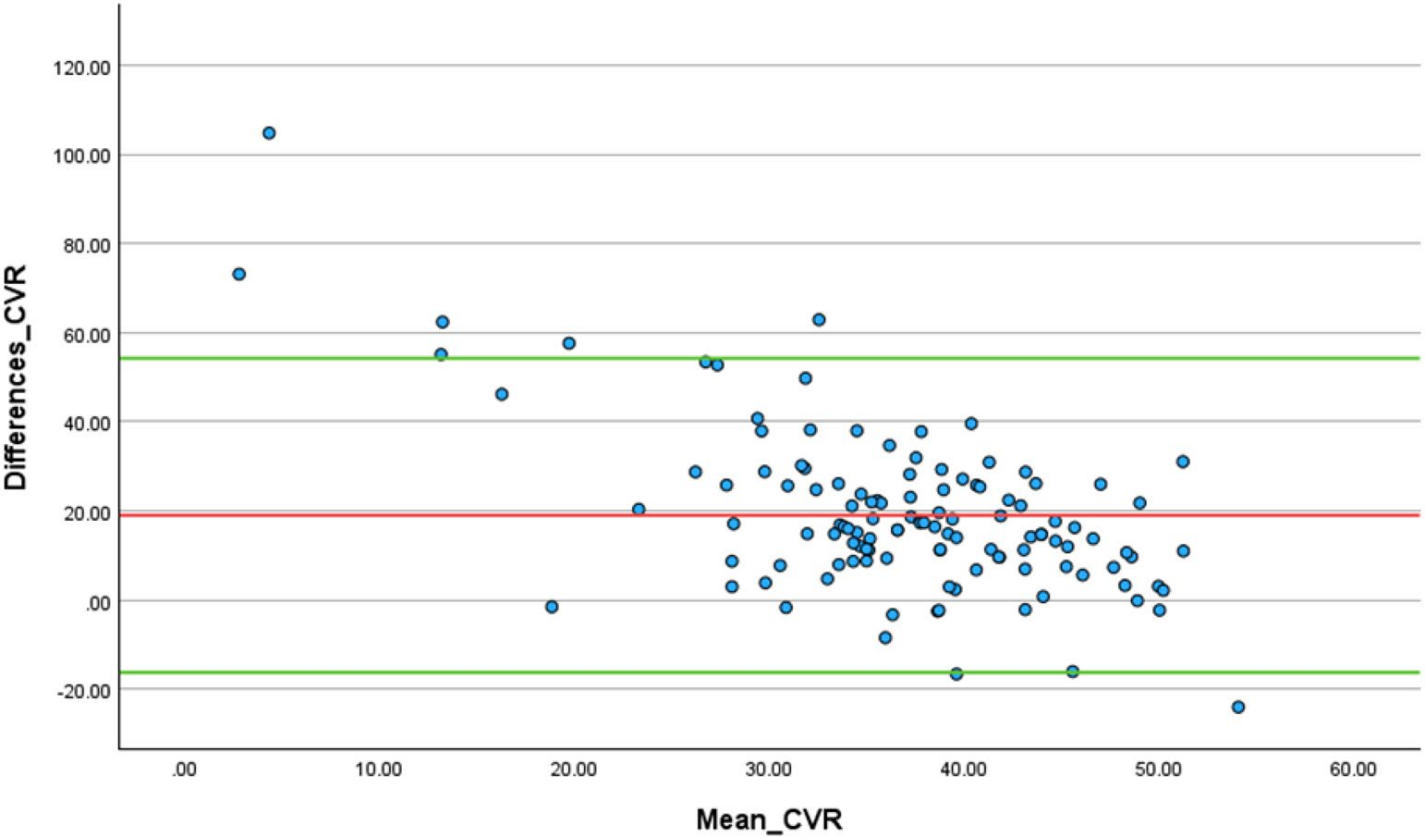
Bland-Altman analysis. The visual plots, with a Mean difference of 18.98 (95% confidence interval (CI): 15.75 to 22.22), show that CVR metrics by breath-holding and BHI are not interchangeable

The measures of CVR by breath-holding were positively correlated with the measures of BHI (r = 0.704, 95% CI 0.598, 0.786, *p* < 0.001), as shown in Table 2. However, a Bland–Altman analysis comparing the level of agreement between breath-holding and the BHI revealed a mean difference of 18.98 (95% CI 15.75, 22.22), indicating that the two methods are not interchangeable due to a significant systematic bias (*p* < 0.001). This finding was ascertained through the visual inspection of the Bland–Altman plot, which showed moderately scattered data points around the mean difference line, indicating significant variability in the agreement between the two metrics (Fig. 1).

**Table 2:** Correlations between CVR, haemodynamic parameters, and cognitive performance

Reactivity metric	Correlation parameter	Spearman’s rho	Significance (2-tailed)	95% confidence intervals (2-tailed)	
				Lower	Upper
CVR (BHI)	Cerebrovas. conductance	0.468	< 0.001	0.312	0.600
	CVR (Breath-holding)	0.704	< 0.001	0.598	0.786
	CVR (hyperventilation)	0.105	0.252	− 0.080	0.283
	Cognitive performance	0.893	< 0.001	0.848	0.925
CVR (Breath-holding)	Cerebrovas. conductance	0.055	0.544	− 0.129	0.236
	Breath-holding index	0.704	< 0.001	0.598	0.786
	CVR (hyperventilation)	0.031	0.735	− 0.153	0.214
	Cognitive performance	0.772	< 0.001	0.686	0.837
CVR (hyperventilation)	Cerebrovas. conductance	0.216	0.017	0.034	0.385
	Cognitive performance	0.017	0.856	− 0.168	0.200
Cerebrovas. conductance	Cognitive performance	0.147	0.106	− 0.037	0.321

CVR: Cerebrovascular reactivity, BHI: Breath-Holding Index, Cerebrovas: cerebrovascular

The associations among the CVR metrics, as well as the trends between these metrics and cerebrovascular conductance, were diverse. There was a significantly positive association between BHI and cerebrovascular conductance (r = 0.468, 95% CI 0.312, 0.600, *p* < 0.001). CVR by breath-holding also showed a positive correlation with cerebrovascular conductance; however, this association was not statistically significant. On the other hand, CVR by hyperventilation showed a significantly positive correlation with cerebrovascular conductance (r = 0.216, 95% CI 0.034, 0.385, *p* = 0.017). Notably, CVR by hyperventilation showed no statistically significant associations with either CVR by breath-holding or BHI (*p* > 0.05, respectively). These findings indicate that the CVR metrics have distinct associations with one another and with cerebrovascular conductance (Table 2).

### CVR metrics, conductance, and cognitive performance

Spearman's correlation analysis revealed strong positive correlations between cognitive performance and both metrics of CVR by breath-holding and BHI (Table 2). In contrast, neither CVR by hyperventilation nor cerebrovascular conductance showed statistically significant correlations with cognitive performance (p > 0.05 for each).

**Fig. 2:**
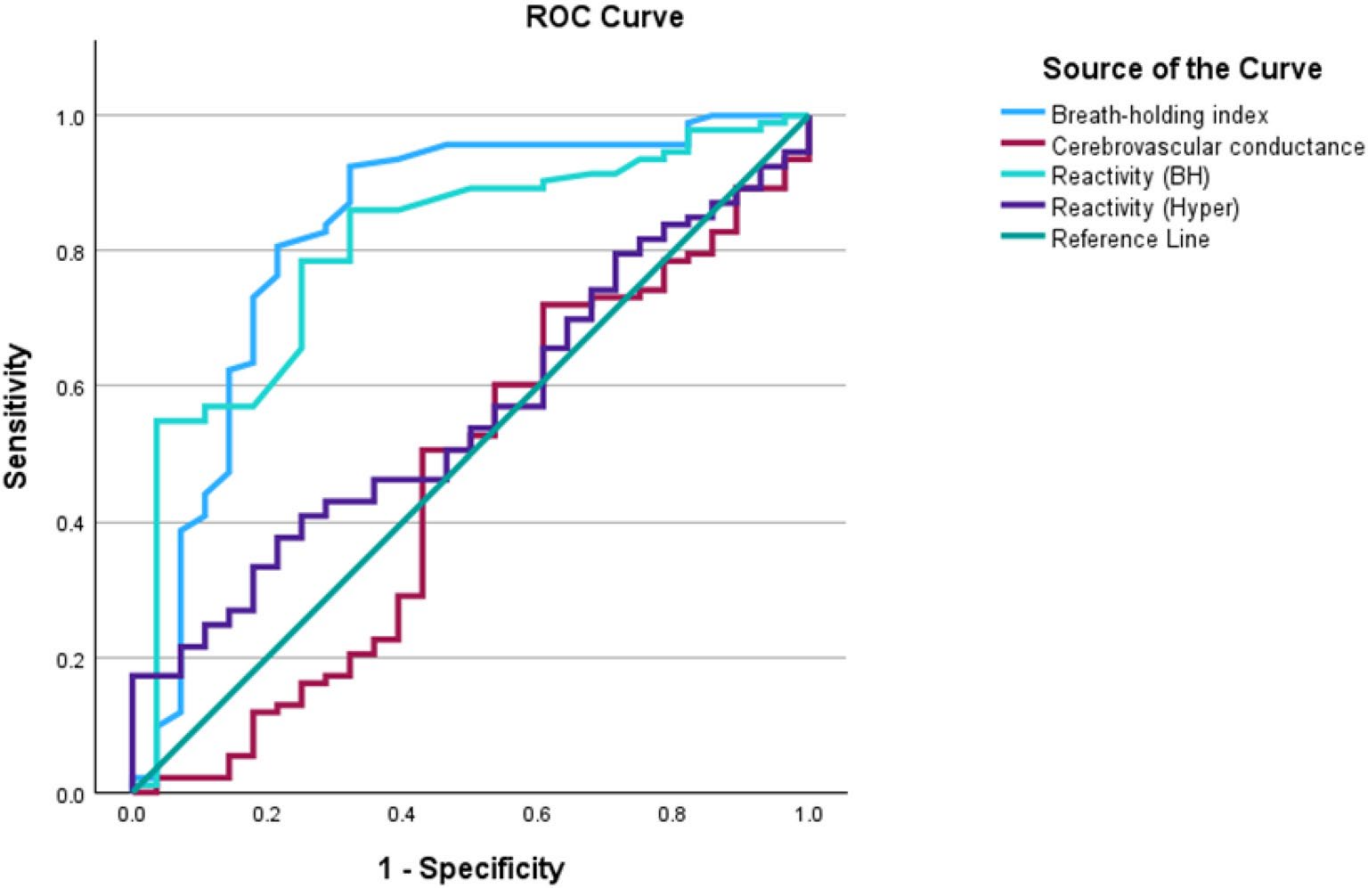
ROC analysis curve. BHI showed the highest accuracy in predicting mild cognitive disorders

**Fig. 3:**
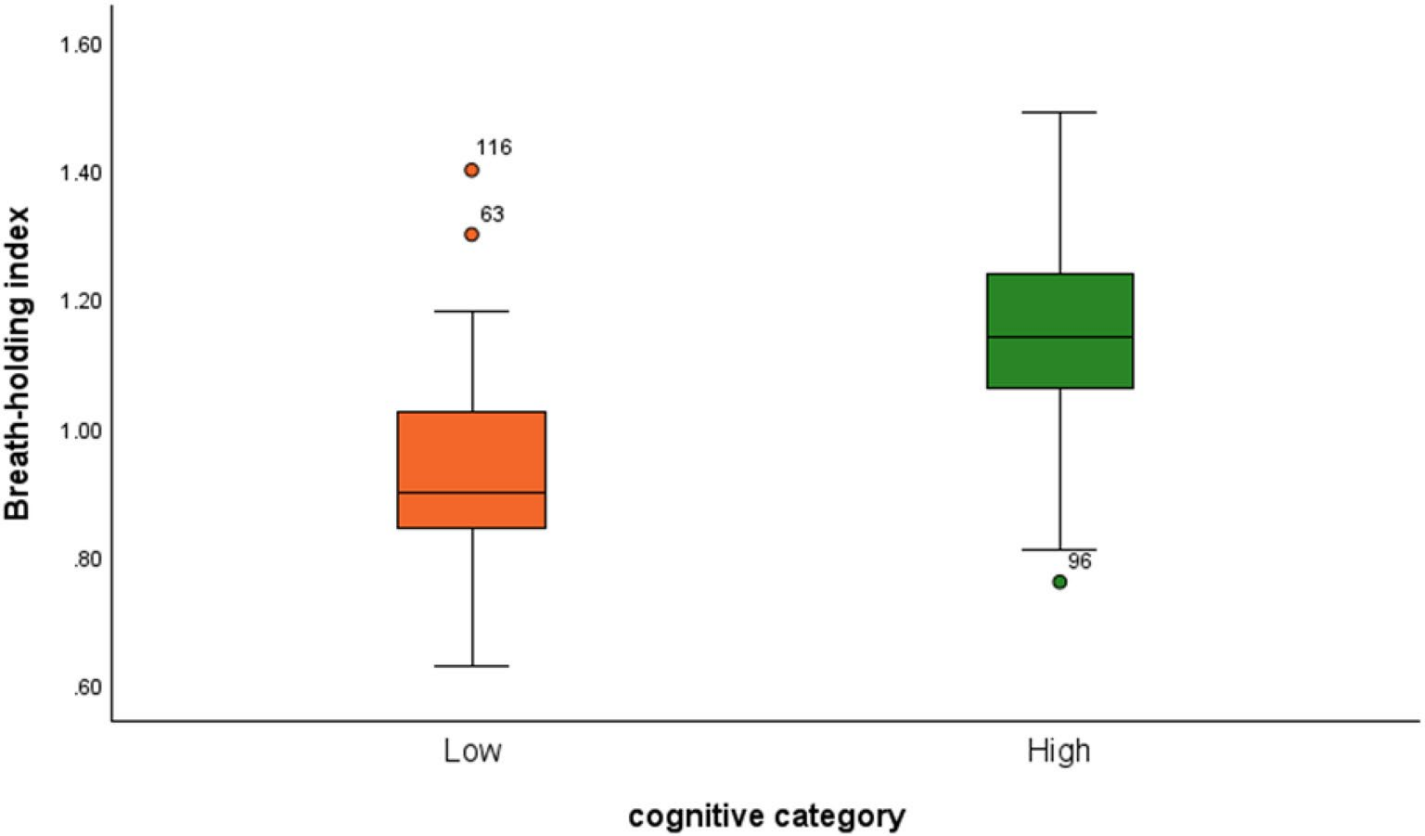
CVR (BHI) and cognitive performance. Participants who recorded low cognitive performance scores (< 26) demonstrated significantly lower BHI scores, *t* (37) = − 5.6, 95% CI: − 0.279, − 0.131, *p* < 0.001, compared to the participants with higher cognitive performances (> 26)

Figure 2 compares the accuracies of CVR metrics and cerebrovascular conductance as predictors of cognitive performance. All three CVR metrics outperformed cerebrovascular conductance. Table 3 records the AUC values, sensitivity, and specificity from ROC analysis in predicting mild cognitive impairment. Using the most accurate CVR metric (BHI), independent t-test statistics showed participants with mild cognitive impairment (n = 28) had significantly lower CVR scores (*t* (37) = − 5.6, 95% CI − 0.279, − 0.131, *p* < 0.001). This was compared to the 94 cognitively normal participants (Fig. 3).

**Table 3:** Area under the ROC curve

Test result variable (s)	Area	Std. Error^a^	Asymptotic Sig.^b^	Asymptotic 95% confidence interval		Sensitivity	Specificity
				Lower bound	Upper bound		
Breath-holding index	0.827	0.052	0.000	0.725	0.930	0.88	0.85
Reactivity (BH)	0.805	0.047	0.000	0.713	0.047	0.80	0.75
Reactivity (Hyper)	0.559	0.057	0.300	0.447	0.057	0.60	0.55
Cerebrovascular conductance	0.462	0.066	0.567	0.332	0.066	0.45	0.50

^a^Under the nonparametric assumption

^b^Null hypothesis: true area = 0.5

### BHI and cognitive performance

As shown in Table 4, model 1, adjusted for age and sex in multiple linear regression analysis, yielded an R^2^ = 0.819. Here, BHI independently predicted total cognitive performance F (3, 128) = 178.224*, β* = 0.912, t = 23.02, *p* < 0.001). Model 2 adjusted for age, sex, and vascular risk factors (smoking, drinking, hypertension, hyperlipidaemia, diabetes mellitus, BMI, and medication use) with R^2^ = 0.860. Here, BHI was shown as a significant predictor of total cognitive performance F (12, 109) = 56.027*, β* = 0.957, t = 24.24, *p* < 0.001).

**Table 4:** Multiple regression models of BHI and cognitive performance

Model		Beta coefficients		t	Sig.
		B	Std. error		
	(Unadjusted model)				
	Breath-holding index	0.895	0.817	21.995	< 0.001
	(Adjusted model)				
1	Breath-holding index	0.912	0.796	23.020	< 0.001
2	Breath-holding index	0.957	0.793	24.243	< 0.001

Model summary^d^									
Model	R	R square	Adjusted R square	Std. error of the estimate	Change statistics				
					R square change	F change	df1	df2	Sig. F change
	0.895^a^	0.801	0.800	1.537	0.801	483.774	1	120	< 0.001
1	0.905^b^	0.819	0.815	1.478	0.018	5.859	2	118	0.004
2	0.928^c^	0.860	0.845	1.351	0.041	3.584	9	109	< 0.001

^a^Predictors: (Constant), Breath-holding index

^b^Predictors: (Constant), Breath-holding index, Sex, Age

^c^Predictors: (Constant), Breath-holding index, Sex, Age, Diabetes, Drinking, PR, Smoking, BMI, DBP, Hyperlipidaemia, Hypertension, SBP, and medication use

^d^Dependent Variable: Total Cognitive performance

### CVR by breath-holding and cognitive performance

Table 5 presents two adjusted models showing the association between CVR by breath-holding and cognitive performance. Both model 1 (R^2^ = 0.466, p = 0.663) and model 2 (R^2^ = 0.518, p = 0.242) had positive coefficients: β = 0.678, t = 10.07, p < 0.001 and β = 0.655, t = 9.63, p < 0.001, respectively. However, the non-significant R^2^ values limit the reliability of this CVR metric. These findings suggest that CVR by BHI is the most reliable predictor of mild cognitive impairment.

**Table 5:** Regression models of CVR by breath-holding and cognitive performance

Model		Beta coefficients		t	Sig.
		B	Std. error		
	(Unadjusted model)				
	CVR (breath-holding)	0.680	0.030	10.16	< 0.001
	(Adjusted model)				
1	CVR (breath-holding)	0.678	0.03	10.068	< 0.001
2	CVR (breath-holding)	0.655	0.03	9.627	< 0.001

Model summary^d^									
Model	R	R square	Adjusted R square	Std. error of the estimate	Change statistics				
					R square change	F change	df1	df2	Sig. F change
	0.680^a^	0.462	0.458	2.528	0.462	103.171	1	120	< 0.001
1	0.683^b^	0.466	0.452	2.540	0.004	0.412	2	118	0.663
2	0.720^c^	0.518	0.465	2.511	0.052	1.307	9	109	0.242

^a^Predictors: (Constant), Reactivity (BH)

^b^Predictors: (Constant), Reactivity (BH), Age, Sex

^c^Predictors: (Constant), Reactivity (BH), Age, Sex, Diabetes, PR, Drinking, Smoking, DBP, BMI, Hyperlipidaemia, Hypertension, SBP, and medication use

^d^Dependent Variable: Total Cognitive performance

### Variabilities in CVR assessment: the 4 cases of BHI

**Fig. 4:**
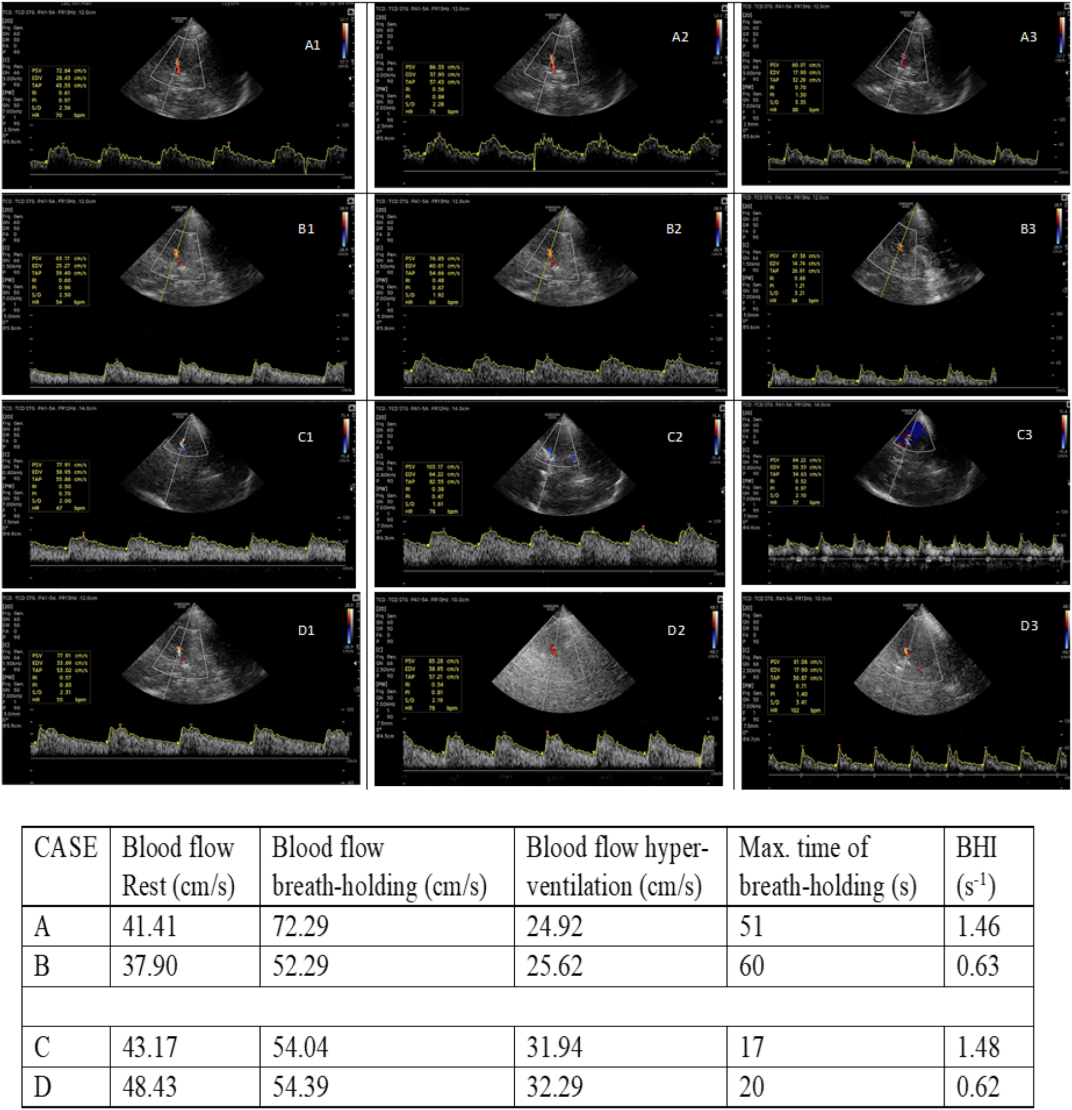
Variabilities in CVR assessment: the 4 cases of BHI. In case A, the participant recorded a high BHI = 1.46 s^−1^ at a maximal tolerable breath-holding duration = 51 s. In case B, the participant recorded a low BHI = 0.63 s^−1^ at a maximal tolerable breath-holding duration = 60 s. In case C, the participant recorded a high BHI = 1.48 s^−1^ at a maximal tolerable breath-holding duration = 17 s. In case D, the participant recorded a low BHI = 0.62 s^−1^ at a maximal tolerable breath-holding duration = 20 s

Figure 4 illustrates the varying CVR responses among four male participants. In Case A, the participant sustained breath-holding for a longer duration, resulting in a significant increase in cerebral blood flow velocity and a higher BHI. In contrast, Case B showed a participant who, despite a similarly prolonged breath-holding duration, did not exhibit significant changes in blood flow velocity and hence recorded a low BHI. Case C involved a participant who experienced rapid and marked increases in cerebral blood flow velocity over shorter durations, resulting in a high BHI. Conversely, Case D showed a participant with minimal changes in blood flow velocity during a short breath-holding period, leading to a lower BHI.

## Discussion

In this study, the three physiologic metrics of CVR assessed via simple breathing techniques exhibited diverse interrelations with cerebrovascular conductance and cognitive performance. TCCD is demonstrated to be a useful and reliable tool for assessing CVR, yielding good to excellent reproducible measures for CVR through breath-holding, hyperventilation, and BHI. The measures of CVR by breath-holding and the BHI were found to be non-interchangeable, despite a significant correlation between the two metrics. All three metrics of CVR outperformed the measures of cerebrovascular conductance as predictors of mild cognitive impairment. Compared to cerebrovascular conductance and the other CVR metrics assessed through breath-holding and hyperventilation, the BHI emerged as the most accurate and reliable predictor of mild cognitive impairments in stroke-free, healthy individuals in their 60 s. The assessment of the BHI effectively accounted for individual physiological time-based variability in CVR responses and was particularly well-suited for evaluating cerebral haemodynamic risks related to cognitive performance. In fact, 23% (of 122) participants who exhibited mild cognitive disorders with low cognitive performance correspondingly recorded lower measures of BHI. The measures of CVR, cerebrovascular conductance, cerebral blood flow and cognitive performance were comparable between males and females.

This study suggests that the cerebral vasomotor response to hypercapnia, as assessed through breath-holding and the BHI, may not directly influence the vasomotor response to hypocapnia induced by hyperventilation. This assertion was corroborated by Daher and Payne [5], who linked the variations in CVR responses induced by hypercapnia and hypocapnia more to the myogenic tone and autoregulatory mechanisms of the underlying vessels being studied than solely based on the vasodilatory stimulus [33, 34]. According to Wei and colleagues [24], arteriolar alterations in vessel radius and the downstream transmission of intravascular pressure influence cerebral blood flow and conductance, which ultimately affects the autoregulatory mechanisms and CVR [25]. Functionally, the increased CVR may serve as a protective mechanism to restore and maintain cerebral blood flow in response to physiological or pathological decreases in arterial pCO_2_ levels [5]. This process maintains adequate perfusion and oxygenation of cerebral tissue, thereby supporting brain health and potentially mitigating cognitive consequences. Consistently, this current study has shown that increased measures of CVR—specifically via breath-holding index and hyperventilation—correspond to increased blood flow and conductance, which is shown in previous studies to improve brain perfusion [16]. The time-based variability observed in CVR measures among individuals suggest that CVR may not be solely influenced by changes in arterial pCO_2_, as previously thought [26], but also significantly by the duration of response to vascular stimuli and the magnitude of blood flow changes (supplementary Fig. 1). Previous studies suggest that factors such as individual variations in vascular tone, baseline cerebral blood flow, and the efficiency of dynamic cerebral autoregulation mechanisms may play crucial roles in determining an individual’s cerebrovascular function [15, 33].

In this study, the average percentage measures of CVR by breath-holding (46.35 ± 7.73), recorded at the 30th second, were found to be closely aligned with the findings of Settakis and colleagues, who reported a mean percentage of 41.4 ± 21.5 in their previous research. In contrast, the current study demonstrated a lower average CVR by hyperventilation (27.32 ± 15.99) compared to the results of Settakis and colleagues, who observed a mean CVR of 38.7 ± 14.5 at the 60th second of hyperventilation. This discrepancy may be partially attributed to the age difference between the study populations. Settakis and colleagues included participants who were relatively younger, with a mean age of 26.3 ± 5.6 years, which may have enabled them to perform hyperventilation more effectively than the older participants in the current study, who had a mean age of 65.34 ± 6.86 years. In a study by Lin and colleagues [27], the assessment of reactivity based on BHI yielded an average measure of 1.40 ± 0.45 among 20 healthy stroke-free control groups. The assessment of BHI in Lin and colleagues' study was restricted to 30 s, which we assert does not fully account for the individuals’ physiologic variabilities, as it overlooks the flow dynamics before and beyond this time threshold. This previous approach of quantifying BHI assumes each participant attains maximal vasodilation at the 30th second [19], which this current study demonstrates is not the case (Fig. 4). In the current study, the average BHI was 1.09 ± 0.17, ranging from 0.63 to 1.49, accounting for time-based variabilities in individuals’ CVR responses, which should not be overlooked. Our study highlights the importance of considering individual physiological differences in cerebrovascular haemodynamic assessments.

It is imperative to consider the existing challenges associated with various assessment techniques and the clinical implications of the metrics derived. For instance, one significant issue with hyperventilation is the feeling of discomfort and the variability in the pace at which participants perform hyperventilation, which can lead to inconsistent outcomes. Differences in breathing rate and depth among individuals can result in varying levels of arterial pCO_2_ reduction, thereby affecting the reliability of the CVR measurements. CVR by breath-holding is traditionally quantified over a restricted duration of 30 s, which can compel individuals to strain themselves to complete the test. Such straining could be very uncomfortable and could yield counterproductive effects. These challenges highlight the need for standardised protocols and tolerable alternative metrics. Our approach offers more consistent and individualised assessments of CVR. Instead of strictly following the conventional 30 s breath-holding or 60 s hyperventilation durations [19], we recommend that clinicians and researchers consider adopting the BHI as a more reliable and robust measure of CVR that can be easily assessed using TCD and now, TCCD. In fact, the observed dissociation between the BHI and standard breath-holding-derived CVR metrics, as demonstrated by the Bland–Altman analysis, indicates that these measures may capture distinct facets of cerebrovascular physiology. While the standard breath-holding-derived CVR typically reflects global vascular responsiveness to hypercapnic stimuli, BHI—particularly when assessed with optimised, individualised approaches—provides a more sensitive index of flow dynamics and subject-specific vasomotor response, capturing subtle variations that broader metrics may overlook [28–30].

The strengths of this study include its utilisation of comprehensive, non-invasive, simple techniques, including breath-holding, hyperventilation, and BHI for assessing CVR. Incorporating TCCD as part of our methodology to perform these assessments highlights significant clinical advantages. TCCD provides a non-invasive, real-time assessment of cerebral blood flow velocity, enabling the precise measurement of CVR and cerebrovascular conductance across different conditions. The ability of TCCD to visualise blood flow dynamics enhances the accuracy of our findings and supports the use of the BHI as a flexible measure of CVR. According to the BHI assessments, it can be inferred that the ability to hold one's breath for a longer duration does not necessarily indicate better neurovascular function. Again, this study factored individual physiological differences based on the duration of response to stimuli into the assessment of CVR by BHI. This approach enabled more personalised assessments, which could enhance the accuracy and applicability of CVR measurements in clinical practice. The clinical significance of BHI lies in its role as a modifiable vascular marker responsive to interventions such as aerobic exercise, dietary changes, and optimal management of vascular risk factors [31, 32]. Accordingly, BHI may serve as both an early biomarker for cerebrovascular dysfunction and a surrogate endpoint for monitoring therapeutic efficacy. Identifying early cerebrovascular dysfunction is paramount because it often precedes clinically overt cognitive decline [17]. By targeting BHI and related vascular indices, it may be possible to prevent or slow the progression from mild cognitive impairment to dementia. This strategy supports the overarching objective of reducing cognitive decline through optimisation of vascular health.

Despite the significant outcomes, the current study has some limitations that must be considered when interpreting the results. The study included only Chinese adult participants, which may limit the generalisability of the findings to other populations. Administering the CVR assessments in a fixed order may introduce systematic or carryover effects that could influence the outcomes, despite the use of washout periods; however, evidence suggests that, with adequate washout intervals, such order effects are unlikely to confound primary CVR measures [33]. Although well-controlled in this study, techniques such as breath-holding and hyperventilation could cause discomfort or anxiety in some patients in a clinical setting, which might influence their performance and the study outcomes. Nevertheless, these factors were controlled among our healthy participants to ensure the accuracy of CVR assessments.

In conclusion, this study establishes TCCD as a reliable and practical modality for assessing CVR through straightforward, non-invasive, and clinically tolerable techniques such as breath-holding, hyperventilation, and, most notably, the BHI. The findings reveal that BHI, measured via TCCD, is the most sensitive and robust metric for predicting mild cognitive impairment, outperforming other CVR and conductance measures. Notably, BHI effectively captures individual physiological variability and provides a comprehensive evaluation of cerebral haemodynamics relevant to cognitive health. These findings support the practical use of TCCD-derived BHI for early cerebrovascular risk screening and personalised neurovascular assessment in healthy older adults.

## Materials and methods

### Study design and participants

To assess CVR and cerebrovascular haemodynamic parameters, 150 stroke-free, healthy Chinese adults (over 45 years of age) of either gender from our ongoing community-based cohort were randomly recruited. The clinical data and MRI images of each participant were reviewed at the University Research Facility in Behavioural and Systems Neuroscience (UBSN) of the Hong Kong Polytechnic University to ascertain participants’ eligibility. Prior to selection for the reactivity tests, all participants underwent a thorough ultrasound examination of the carotid arteries to exclude the presence of haemodynamically significant stenosis. Selected participants were deemed eligible following a subsequent successful demonstration of the breath-holding and hyperventilation manoeuvres and the test procedures described to them in a demonstration room. We excluded participants with a history of stroke, individuals on vasodilatory medications, particularly those treated with beta-blockers, people living with dementia, substance abuse, neuropsychiatric conditions, brain tumours, or injury, as well as those with living neurodegenerative diseases such as Parkinson’s or Alzheimer’s. Individuals currently prescribed beta-blockers, specifically β1-adrenergic receptor antagonists such as metoprolol and nebivolol, were excluded because evidence susgests that these medications directly affect cerebral vasomotor function and cerebrovascular reactivity, potentially confounding the assessment of primary outcomes [34, 35]. All individuals with poor trans-temporal acoustic windows were also excluded. The Institutional Review Board of the Hong Kong Polytechnic University approved our protocol (Ethics Approval: **HSEARS20240318007**). The research protocol aligns with the ethical principles outlined in the 1975 Declaration of Helsinki. All participants gave written informed consent.

### Sample size and power justification

Sample size determination was guided by an a priori power analysis conducted using G*Power 3.1 [36, 37]. For correlation analyses examining the association between CVR metrics, conductance, and cognitive performance, an expected medium effect size (r = 0.3) was adopted based on prior literature [3]. To achieve 90% power at a significance level of α = 0.05 (two-tailed), a minimum of − 110 participants was required. For multiple linear regression analyses evaluating each CVR metric’s predictive value on cognition while adjusting for nine covariates, a medium effect size (f^2^ = 0.15) was assumed. With 10 predictors total, the same α and power thresholds required approximately 118 participants. Thus, a sample of 122 participants is adequate for detecting meaningful effects in subsequent analyses.

### Cognitive assessment

This study employed the validated Hong Kong version of the Montreal Cognitive Assessment (HK-MoCA), which was modified to account for the effects of educational background and cultural differences [38]. All cognitive function assessments were performed prior to the CVR assessments. To minimise potential fatigue or physiological effects, participants rested for at least 20 min between the completion of cognitive testing and the initiation of CVR procedures. There are seven domains in HK-MoCA, namely visuospatial, executive, naming/language, attention, alertness, abstract thinking and memory functions. The scores, ranging from 0 to 30, were modulated by the number of years of education. It served as a reference for potential cognitive impairment in participants scoring below 26. For the purposes of analysis, participants with high scores > 26 were designated to be cognitively normal, while those with low scores < 26 were designated to have a mild cognitive impairment or disorder. Each participant was assessed by a trained neurologist.

### TCCD scanning and protocol

All ultrasound investigations were carried out in a quiet ultrasound laboratory by an experienced sonographer (tester), who was blinded to the clinical data. Each participant refrained from consuming alcoholic or caffeinated beverages and engaging in rigorous exercise for at least 24 h prior to their appointment day. Ultrasound scanning was performed and completed in one day, with each participant lying comfortably in the supine position. The assessments were performed using the Samsung RS85 ultrasound machine (Samsung Medison Co., Ltd., Seoul, Republic of Korea), equipped with a 1–5 MHz phased array transducer (power output = 90%, frequency = general preset, dynamic range = 50, frame average = 8, scan area = 100%, focus = 1, and gain = 50) for all vascular haemodynamic assessments. The Samsung RS85 ultrasound machine is specifically equipped with TCCD software. The TCCD preset is capable of handling continuous velocity recordings while capturing desired snapshots of waveforms at specific windows. This functionality enables the tester to perform cerebral blood flow assessments using measures that have been shown to be comparable to those of traditional TCD devices [13].

### Scanning technique

In a good transtemporal acoustic window [39], the middle cerebral arteries (MCAs) were the cerebral vessels of interest. A 1.6 MHz hand-held probe was used for insonation throughout the entire study. For each insonated MCA, the blood flow velocity is recorded by averaging the two closest values obtained from standardised regions of interest: the proximal segment, the midpoint, and the distal segment at the bifurcation of the MCA [13]. The MCAs are easily identified in brightness (B) mode using the hypoechogenic, butterfly-shaped mesencephalic brain stem structures, surrounded by a hyperechoic, star-shaped basal cistern, as landmarks. The blood flow and direction, as well as spectral waveform patterns, were consecutively displayed using the colour and spectral Doppler modes, respectively. The scanning parameters for brightness mode, colour Doppler, and spectral Doppler were optimised in accordance with the user-defined optimised preset under the TCD application available on the RS85 Samsung ultrasound machine. The scanning protocol is detailed in a previous study [13]. With a sweep speed of 5 s per screen, the automated spectral analysis function records the average velocity by enveloping 5 to 7 stable waveforms, capturing their peak values. In this study, MCA velocities were recorded from a single window with stable waveforms.

### Cerebrovascular conductance and flow assessment

Prior to the CVR assessment, the mean arterial pressure (MAP) was measured from systolic and diastolic arterial blood pressures. We utilised a continuous non-invasive blood pressure monitoring system, specifically employing an upper arm cuff method corroborated with a reliable electrosphygmomanometry (Omron, HEM-8717) [40], as described by the American Heart Association [41]. Participants rested in the supine position for at least five minutes prior to measurement to minimise any physiological fluctuations due to activity or stress [42]. The mean of triplicate measures of blood pressure taken at 1 min intervals was recorded. The cerebral mean blood flow velocity (cm/s) via the MCA was measured at rest. Cerebrovascular conductance—a functional measure of cerebral blood flow relative to mean arterial pressure was calculated as the Vmean/MAP ratio [25].

### Reproducibility of CVR assessments using TCCD

Breath-holding and hyperventilation, per Settakis et al., were used as physiological stimuli to assess the transient blood flow changes for hypercapnia and hypocapnia, respectively, without CO_2_ or acetazolamide [19]. This approach stemmed from the fact that hypercapnia (an increase in arterial carbon dioxide end-tidal pressure (pCO2)) is induced in breath-holding, and hypocapnia (decrease in pCO2) is induced in hyperventilation. As adopted by Lasek-Bal et al., measurements of CVR were performed within the left MCA, as there were no significant differences in blood flow velocity between the contralateral sides [43]. At each point of measurement, the automated spectral analysis function records the maximum blood flow velocity by enveloping 5–7 stable waveforms. The sequence of CVR assessments (breath-holding, hyperventilation, and BHI) was fixed across all participants with standardised washout periods between tests to minimise potential carryover effects.

### CVR by breath-holding

In a resting state of about 5 min, the baseline mean flow velocity is recorded with each participant comfortably lying in the supine position. Afterwards, the participant is instructed to hold their breath for 30 s. While monitoring the continuous waveforms, the maximum velocity at the 30th second is captured and recorded using the automated spectral analysis function. Both the baseline velocity at rest (MCA*rest*) and the maximum velocity at the 30th second (MCA*test*) are recorded for calculations. The CVR during breath-holding is calculated based on the percentage change in cerebral mean blood flow velocity, using the formula:

$${\text{CVR }}\left( {{\text{breath}} - {\text{holding}}} \right)\;{ = }\;\frac{{{\text{MCA}}test - {\text{MCA}}rest}}{{{\text{MCA}}rest}} \times 100$$

where MCA_test_ is the MCA mean blood flow velocity measured for 30 s of breath-holding, and MCA_rest_ denotes the blood flow velocity at rest.

### CVR by hyperventilation

Following a 5 min rest to ensure flow dynamics are restored to normal [20], the participant, lying in a supine position, proactively performs hyperventilation for 60 s [19]. Resting breathing rates were measured prior to the hyperventilation test to ensure participants were in baseline respiratory conditions, and the hyperventilation rate was individually adjusted based on these measurements. The hyperventilation technique ensured that each individual achieved a sufficient increase in breathing rate (25–30 breaths per minute (bpm)), depending on baseline breathing rate. For those with baseline breathing rates of 12–15 breaths per minute, hyperventilation was conducted at a rate of at least 25 bpm. Participants with baseline rates of 16–20 bpm were instructed to hyperventilate at a rate of at least 30 bpm. This approach ensured that each participant achieved a sufficient increase in breathing rate to induce the desired physiological effects for the study. This protocol was designed based on validated methods that reliably induce hypocapnia through controlled hyperventilation [19, 24, 30]. The CVR during hyperventilation is calculated based on the percentage change in cerebral blood flow using the formula:

$$\begin{gathered} {\text{CVR}}\;\left( {{\text{hyperventilation}}} \right)\; = \;\frac{{{\text{MCA}}rest - {\text{MCA}}test}}{{{\text{MCA}}rest}} \times 100 \hfill \\ \hfill \\ \end{gathered}$$

where MCA_test_ is the MCA mean blood flow velocity measured after 60 s of hyperventilation, and MCA_rest_ denotes the blood flow velocity at rest.

### CVR by BHI

Given that changes in the MCA blood flow may persist for 3 min after performing hyperventilation [20], participants rested for 5 min. After a normal resting state, the participant is now instructed to perform another breath-holding, but this time, they are not restricted to 30 s of breath-holding as done in previous studies [27]. Participants cease their breath for as long as they can hold **without straining** themselves. While monitoring the continuous increases in flow velocities, the maximum value is captured when participants press a button indicating their inability to continue breath-holding. The time captured is considered the maximum tolerable breath-holding duration for that participant. This approach captures individual physiologic variabilities, as depicted by the four cases illustrated in Supplementary Fig. 1. CVR measured by BHI was quantified based on the percentage change in blood flow velocity over the maximum tolerable breath-holding duration (time in seconds) elapsed, according to the formula:

$$BHI\, = \,\frac{{\frac{{{\text{MCA}}test - {\text{MCA}}rest}}{{{\text{MCA}}test}} \times 100}}{Max.\,Breath\, - \,holding\,time}$$

where MCA_test_ is the mean blood flow velocity in the MCA measured during breath-holding, MCA_rest_ denotes the blood flow velocity at rest and Max breath-holding time is the maximum tolerable duration (in seconds) of breath hold.

### Intra-tester reliability of using TCCD for CVR metrics

The intra-tester reliability of using TCCD to quantify CVR metrics was evaluated among 50 participants using the Intraclass Correlation Coefficient (ICC) to assess the reproducibility of the tests. This sample size was chosen to provide robust and precise estimation of the ICC, based on current recommendations for reliability studies, which recommend a minimum of 30–50 subjects for adequate statistical precision [45]. The measures of CVR through breath-holding, hyperventilation, and the BHI were conducted twice for the same participant by the same tester. ICC estimates, along with their 95% confidence intervals, were calculated based on average measures of consistency (k = 2) using a two-way mixed-effects model in the SPSS statistical package (version 29.0). The results were as follows: CVR by breath-holding had an ICC of 0.864 (95%CI 0.76, 0.923), CVR by hyperventilation had an ICC of 0.856 (95%CI 0.747, 0.918), and BHI had an ICC of 0.907 (95%CI 0.836, 0.946).

### Data collection and covariates

We obtained complete data on transcranial ultrasound-detected haemodynamic variables, including CVR during breath-holding, hyperventilation, and BHI. Other haemodynamic parameters, including cerebrovascular conductance, were also sampled using TCCD assessments. Baseline variables, including age, gender, BMI, blood pressure, mean arterial pressure, and heart rate, as well as participants' status regarding hypertension, diabetes, hyperlipidaemia, smoking, and alcohol consumption, were recorded.

### Statistical analysis

Statistical analysis was performed using the SPSS 29.0 software package (IBM SPSS Statistics, Inc.). The mean values with standard deviation (SD) or the median with interquartile range were presented for continuous variables. The frequencies and percentages of categorical variables were also presented. Spearman’s correlation coefficients were employed to assess relationships between CVR techniques and cerebrovascular conductance, as well as cognitive performance measures. The area under the curve (AUC) values were obtained through a receiver operating characteristic (ROC) curve analysis to evaluate how each of the three metrics of CVR and cerebrovascular conductance performed in predicting cognitive performance outcomes. We used multiple regression analysis to explore the independent association between significant CVR measures and cognitive performance measures. The association was adjusted for age and sex under Model 1; then adjusted for age, gender, and vascular risk factors (smoking, drinking, hypertension, hyperlipidaemia, diabetes mellitus, and BMI and medication use) in Model 2. Statistical significance was attained at p-value < 0.05. The Bland–Altman test was further used to determine how CVR measured by breath-holding and BHI agree with each other.

## Data Availability

The data supporting this study's findings are not publicly available because they contain information that could compromise the privacy of research participants. However, upon reasonable request, the corresponding author, [X.C], at fiona.chen@cpce-polyu.edu.hk, can make these data available.
